# The complete plastid genome of *Delonix regia* (Hook.) Raf. (Leguminosae)

**DOI:** 10.1080/23802359.2020.1715886

**Published:** 2020-01-22

**Authors:** Qiubiao Zeng, Qiang Lai, Shiran Gu, Zhonghui Ma

**Affiliations:** a National Demonstration Center for Experimental Plant Science Education, College of Agriculture, Guangxi University, Nanning, China;; b Key Laboratory of Plant Resources Conservation and Sustainable Utilization, South China Botanical Garden, Chinese Academy of Sciences, Guangzhou, China;; c University of Chinese Academy of Sciences, Beijing, China

**Keywords:** Caesalpinioideae, chloroplast genome, Fabaceae, Madagascar, phylogeny

## Abstract

*Delonix regia*, a plant species of the legume family native to Madagascar, has been widely cultivated in the tropical and subtropical regions as an ornamental tree due to its remarkable showy orange-red flowers over summer. Here we report for the first time the complete plastid genome of this species, which has a typical circular structure with a total length of 162,756 bp and contains two inverted repeat regions (IRs, 25,544 bp), a large single copy region (LSC, 92,490 bp), and a small single copy region (SSC, 19,178 bp). The phylogenetic analysis based on the complete plastome sequences of this species and those of the related species from GenBank strongly suggested that *D. regia* is nested in the subfamily Caesalpinioideae and is sister to a clade consisting of *Erythrophlium fordii* and the old-sense Mimosoideae.

*Delonix regia* (Hook.) Raf., with showy orange-red flowers in the summer, is the best known species of the small genus *Delonix* with 11–13 species in the legume family (Babineau and Bruneau 2017). Originated in Madagascar, it is now very popular in the tropical and subtropical regions of Asia, Australia, Africa and the New World. Here, we generated the complete plastid genome sequence of *D. regia* (GenBank accession number: MN893243) to provide genetic and genomic information to promote its systematic research and garden utilization.

The leaf tissues were collected in South China Botanical Garden, Guangzhou, China (113.369221E, 23.181119 N). The specimens (vouchers: LaiQ083) were deposited in the herbarium of South China Botanical Garden (IBSC). The total genomic DNA was extracted by a modified CTAB method (Doyle and Doyle 1987). After fragmented the isolated total genomic DNA into a library of 300–500 bp, the paired-end sequences in length of 150 bp were generated with Illumina (HiSeq X-Ten) at Beijing Genomics Institute (BGI) in Wuhan, China. We assembled the plastome by GetOrganelle pipeline (Bankevich et al. 2012; Langmead and Salzberg 2012; Wick et al. 2015; Jin et al. 2018), and used Plastid Genome Annotator (PGA) (Qu et al. 2019) and Geneious (Kearse et al. 2012) to annotate and align the complete plastome. The annotated plastome has been deposited in GenBank (accession number: MN893243). We downloaded 15 plastid genome data of related species in the tribe Ormosieae, Dalbergieae, Caesalpinieae, Erythrophleeae, Adenanthereae, Parkieae, Desmantheae, Cassieae, and Ceratonieae from GenBank to reconstruct the phylogenetic position of *D. regia* (Figure 1). We aligned the data matrix using MAFFT (Katoh and Standley 2013) with default parameters. The phylogenetic relationship was estimated using the Maximum Likelihood method by RAxML-HPC2 on XSEDE at CIPRES Science Gateway (Miller et al. 2010) with models recommended by ModelFinder (Kalyaanamoorthy et al. 2017) based on a data matrix of complete plastome sequences. The branch supports were estimated using 1000 replicates of bootstrap.

**Figure 1.**
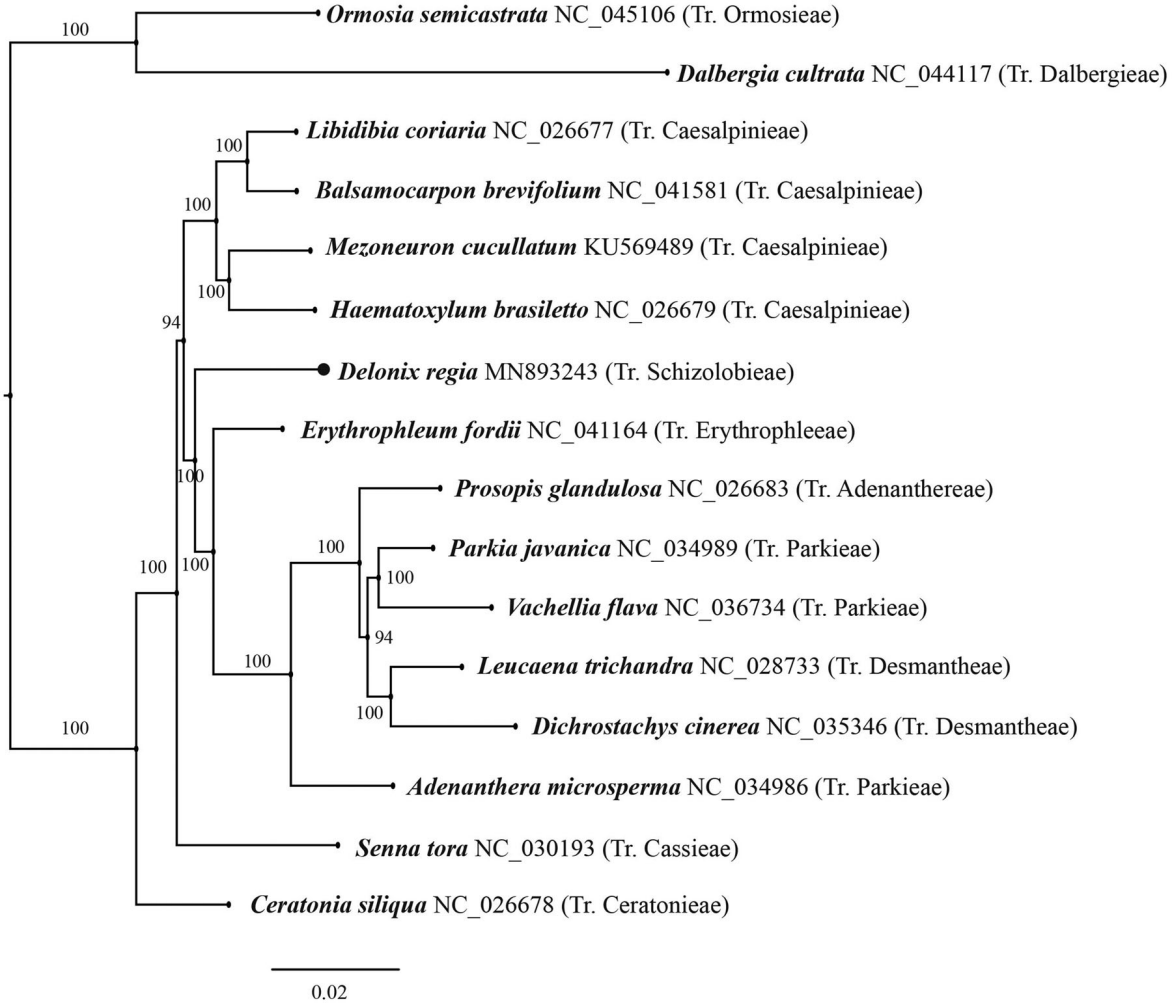
The maximum-likelihood (ML) phylogenetic tree based on the complete plastid genome sequences. Numbers near the branches are bootstrap support values based on 1000 replicates.

The complete plastid genome of *Delonix regia* was 162,756 bp in length and showed a typical quadripartite structure: a large single copy (LSC) region of 92,490 bp and a small single copy (SSC) region of 19,178 bp, respectively. These two regions were separated by two inverted repeat regions (IRa and IRb), each of 25,544 bp in length. We recovered a total of 126 functional genes, including 85 protein-coding genes, 37 tRNA genes and 4 rRNA genes. The overall GC content was 35.7%. The phylogenetic analysis indicates that *D. regia* is nested within the subfamily Caesalpinioideae and is sister to a clade consisting of *Erythrophleum fordii* Oliv. and the old-sense Mimosoideae clade (Figure 1). The complete plastome sequence of *D. regia* will help for the conservation and garden utilization of this species as well as for the phylogenetic studies of Leguminosae.
